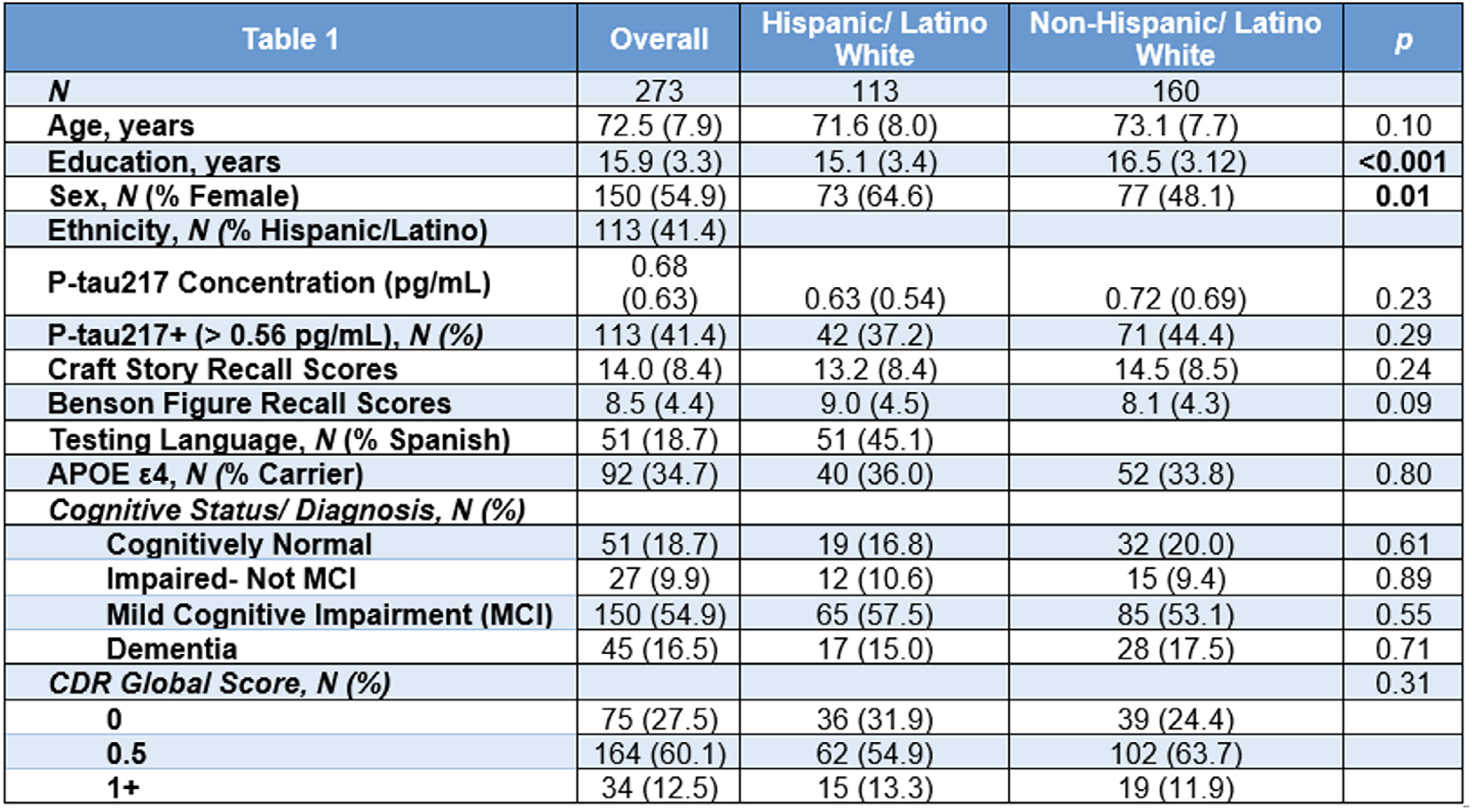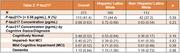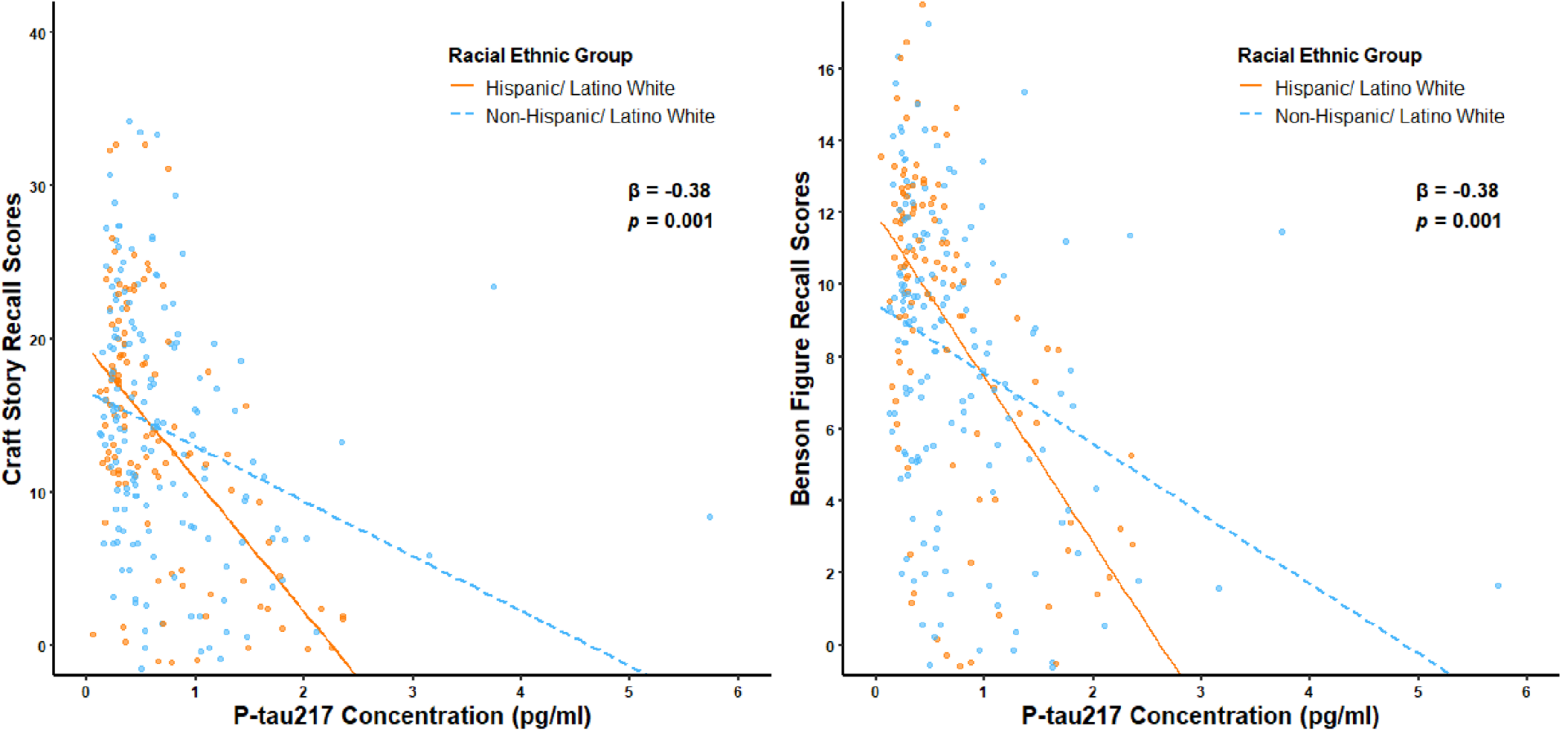# Hispanic/Latino ethnicity moderates the plasma P‐tau217 and memory relationship in older adults across the cognitive continuum

**DOI:** 10.1002/alz.089278

**Published:** 2025-01-09

**Authors:** Shannon Y. Lee, Olivia M Emanuel, Emily F Matusz, Jessica Bove, Jacob Fiala, Franchesca Arias, Shellie‐Anne Levy, Michael Marsiske, Monica Rosselli, Rosie E Curiel, David A. Loewenstein, Ranjan Duara, Idaly Velez‐Uribe, Warren W Barker, David Vaillancourt, Melissa J Armstrong, Glenn E. Smith, Breton M. Asken

**Affiliations:** ^1^ University of Florida, Gainesville, FL USA; ^2^ 1Florida Alzheimer's Disease Research Center, Miami, FL USA; ^3^ 1Florida Alzheimer's Disease Research Center, Gainesville, FL USA

## Abstract

**Background:**

Plasma P‐tau217 demonstrates strong sensitivity and specificity for Alzheimer’s disease (AD) neuropathological change, but existing studies are often ethnically homogeneous. Assumptions about how well plasma P‐tau217 relates to key AD‐related cognitive outcomes like memory may not generalize. We assessed whether the association between plasma P‐tau217 and memory depended on ethnicity in a sample of Hispanic/Latino and non‐Hispanic/Latino White older adults.

**Method:**

We studied 273 1Florida ADRC participants with normal cognition, mild cognitive impairment (MCI), or dementia (Table 1). All participants had plasma samples analyzed for P‐tau217 (ALZPath) and neuropsychological assessments. Using Pearson’s correlations, we first evaluated the bivariate association between P‐tau217, Craft Story verbatim recall (verbal memory), and Benson Figure recall scores (visual memory) overall and stratified by ethnicity. To determine whether ethnicity moderated the effects of P‐tau217 on memory, we used multiple linear regression to assess the interaction of P‐tau217 and ethnicity (Hispanic/Latino vs. non‐Hispanic/Latino) controlling for age, sex, education, and testing language. Post hoc sensitivity analyses separately evaluated individuals below and above internal Aβ PET‐derived P‐tau217 cutoffs for “AD positive” concentration.

**Result:**

Higher P‐tau217 was significantly associated with worse verbal (r= ‐0.38, p < 0.001) and visual memory (r= ‐0.31, p < 0.001) overall and separately in Hispanic/Latino (r= ‐0.56, r= ‐0.57; p’s <.001) and non‐Hispanic/Latino White participants (r= ‐0.30, r = ‐0.34; *p’s* < 0.001). Ethnicity significantly moderated P‐tau217 associations with verbal (β= ‐0.38, p= 0.001) and visual memory (β = ‐0.38, p= 0.001). The negative relationship between P‐tau217 and memory was significantly stronger for Hispanic/Latino than non‐Hispanic/Latino participants. P‐tau217 and demographic covariates explained more variance in verbal (R2= 0.39) and visual memory (R2= 0.37) for Hispanic/Latino than non‐Hispanic/Latino Whites (R2= 0.16 and R2= 0.19, respectively). Post hoc analyses suggested these findings were driven by individuals on the AD continuum.

**Conclusion:**

AD pathology, measured via plasma P‐tau217, may more strongly relate to memory function in Hispanic/Latino than non‐Hispanic/Latino Whites. Findings have implications for the potential roles of co‐pathology or unexplored risk factors in explaining memory loss beyond AD across demographic subgroups. Ethnically representative studies will improve translation of AD biomarkers and inform anticipated AD‐directed treatment effects.